# Hypothermic Perfusion Modifies the Association Between Anti-LG3 Antibodies and Delayed Graft Function in Kidney Recipients

**DOI:** 10.3389/ti.2023.10749

**Published:** 2023-02-20

**Authors:** Habib Mawad, Louis Pinard, Samar Medani, Miguel Chagnon, Julie Boucquemont, Julie Turgeon, Mélanie Dieudé, Katia Hamelin, Annie Karakeussian Rimbaud, Ali Belayachi, Bing Yang, Suzon Collette, Lynne Sénécal, Bethany J. Foster, Marie-Josée Hébert, Héloïse Cardinal

**Affiliations:** ^1^ Research centre, Centre hospitalier de l’Université de Montréal (CRCHUM), Montreal, QC, Canada; ^2^ Department of Mathematics and Statistics, Université de Montréal, Montreal, QC, Canada; ^3^ Montreal Children’s Hospital, McGill University, Montreal, QC, Canada; ^4^ Canadian Donation and Transplantation Research Program, Edmonton, AB, Canada; ^5^ Héma-Québec, Québec, QC, Canada; ^6^ Department of Medicine, Université de Montréal, Montréal, QC, Canada; ^7^ Hôpital Maisonneuve-Rosemont, Montreal, QC, Canada

**Keywords:** delayed graft function, hypothermic perfusion, kidney allograft, ischemia reperfusion injury, autoantibodies, anti-LG3

## Abstract

We previously reported associations between autoantibodies to the LG3 fragment of perlecan, anti-LG3, and a higher risk of delayed graft function (DGF) in kidney transplant recipients. Here, we aimed to determine whether some factors that modulate ischemia-reperfusion injury (IRI) can modify this association. We performed a retrospective cohort study in kidney transplant recipients in 2 university-affiliated centers. In 687 patients, we show that high pre-transplant anti-LG3 are associated with DGF when the kidney is transported on ice (odds ratio (OR): 1.75, 95% confidence interval 1.02–3.00), but not when placed on hypothermic perfusion pump (OR: 0.78, 95% CI 0.43–1.37). In patients with DGF, high pre-transplant anti-LG3 are associated with a higher risk of graft failure (subdistribution hazard ratio (SHR): 4.07, 95% CI: 1.80, 9.22), while this was not the case in patients with immediate graft function (SHR: 0.50, 95% CI 0.19, 1.29). High anti-LG3 levels are associated with a higher risk of DGF in kidneys exposed to cold storage, but not when hypothermic pump perfusion is used. High anti-LG3 are also associated with a higher risk of graft failure in patients who experience DGF, a clinical manifestation of severe IRI.

## Graphical Abstract

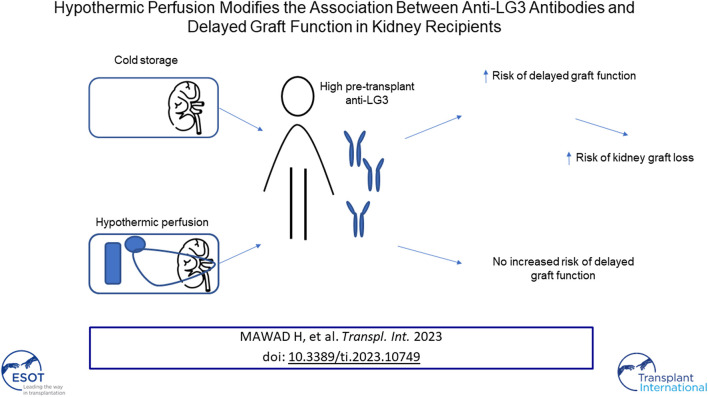

## Introduction

Ischemia-reperfusion injury (IRI) is common in solid organ transplantation. In kidney transplants, severe IRI can result in delayed graft function (DGF). DGF is generally associated with poor long-term renal function and survival (1), although this is not always the case. Some clinical factors such as young donor age or donation following cardiocirculatory arrest are associated with lesser adverse impact of DGF on long-term graft outcomes (2,3), which may be due to the association these factors have with preservation of the kidney microvasculature (4). Mounting evidence indeed suggests that the intensity of peritubular capillary (PTC) injury is a major predictive factor of long-term renal dysfunction after IRI in native and transplanted kidneys (4–8).

Many animal studies have shown that IRI favors the exposure of cryptic autoantigens, which can set in motion autoantibody-dependent tissue injury (9–12). Naturally occurring autoantibodies, such as the ones targeting angiotensin II type 1 receptor (AT1R), vimentin, apoptotic cells and the perlecan fragment LG3 are likely aimed at favoring the clearance of dead cells at sites of injury (10,13–16), but can be involved in tissue injury, especially in the presence of IRI. Our group has also shown in animal models of renal IRI or aortic allogeneic transplantation, that anti-LG3 antibodies prompt complement activation and microvascular rarefaction, but again only in the presence of IRI (10,17). In kidney transplant patients, we found an association between high anti-LG3 levels at the time of transplantation and an increased risk of DGF. We also reported an association between high pre-transplant anti-LG3 and lower estimated glomerular filtration rate (eGFR) 1 year post-transplant, but only in those who experienced DGF. Taken together, these data suggest that IRI is a permissive factor for anti-LG3 to participate in allograft injury. If this is the case, one could hypothesize that factors that are associated with protection from IRI could modify the association between anti-LG3 and DGF. The use of hypothermic perfusion machine rather than ice storage has been associated with a lower risk of DGF (18) and preservation of endothelial function and renal microcirculation in solid organ transplantation (19).

Here, our aim was to assess whether the association between anti-LG3 antibodies and DGF is modified by the use of hypothermic machine perfusion and whether DGF, as a proxy for the severity of IRI, modifies the association between anti-LG3 and allograft survival.

## Materials and Methods

### Patients and Setting

We performed a retrospective cohort study using the University of Montreal Renal Transplant Biobank. From 1st July 2008 to 31st December 2016, consecutive patients undergoing kidney transplantation at two Canadian, university-affiliated hospitals were entered into a clinical and biological database after providing written informed consent. ABO-incompatible transplantations and transplantations crossing pre-transplant DSA are not performed in either center. Serum samples from participants were collected immediately prior to transplantation and banked at −80°C for subsequent analyses. Clinical information was collected prospectively and supplemented retrospectively by chart review as needed. If a patient had more than one kidney transplantation recorded in the database, only the most recent transplant was included. Patients with past or simultaneous non-renal solid organ transplants were excluded. All patients included in the present study were different from those included in our previous publication on rejection (17), and 172 patients were included in our previous work on delayed graft function (10). The project was approved by the local ethics review board of the Centre Hospitalier de l’Université de Montréal (project numbers 14.169 and 16.204).

### Measurements

#### Exposures and Outcomes

For our first aim, the outcome was the occurrence of DGF, which was defined as the need for dialysis in the first post-transplant week, failure of serum creatinine to decrease by more than 10% within the first 3 post-operative days or serum creatinine above 250 µmol/L on post-operative day 5 with evidence of acute tubular necrosis on the allograft scintigraphy (1,10). The exposure, or main independent variable of interest, was high pre-transplant anti-LG3 antibody levels. High anti-LG3 was defined as a value in the highest quartile of the distribution (17). Anti-LG3 was measured with a locally-developed enzyme-linked immunosorbent assay (17). As potential interactions between anti-LG3 levels and ischemia/endothelial injury were suggested by our prior animal studies (17), we defined, *a priori*, use of hypothermic perfusion machine as an effect modifier to be tested. In the province of Quebec, the only pump available in the field of transplantation is hypothermic perfusion with LifePort® devices, which do not provide oxygenation. There is no protocol in place guiding the use of perfusion devices and the decision to use them is hence left to the discretion of the transplant surgeon recovering and/or transplanting the kidney.

For the second aim, the outcome was kidney allograft survival, which was defined as the time elapsed between transplantation and graft failure, either return to dialysis or retransplantation. Death with a functioning graft was taken into account as a competing risk for kidney graft failure. The exposure was high pre-transplant anti-LG3 antibodies, as defined above. The effect modifier was DGF, as defined above. Covariates for all models were selected on the basis of their previously reported associations with DGF or kidney graft survival (20–22). These included both recipient and donor characteristics. Recipient variables included age, sex, race, cause of chronic kidney disease (diabetes, vascular/hypertension, autoimmune and other), time on dialysis before transplantation, height and weight, diabetes, history of cardiovascular disease, smoking status (active, past, never smoker), cytomegalovirus serostatus, pre-transplant and peak panel reactive antibodies, previous transplantations, transfusions, pregnancies, induction (basiliximab as the standard protocol, thymoglobulin with or without intravenous immunoglobulins which are reserved for highly sensitized patients), statin and renin-angiotensin system blockers use on admission. Donor variables included age, sex, height and weight, stroke as cause of death, cytomegalovirus serostatus, history of hypertension, diabetes, vascular disease, terminal creatinine, and number of HLA mismatches with the recipient. Recipient age was the only variable with an *a priori* association with the exposure, anti-LG3 (17).

### Statistical Analyses

Continuous variables are reported as means and standard deviations or medians and interquartile ranges, depending on their distribution. Categorical variables are summarized as numbers and proportions. We analyzed between-group crude differences in non-normally distributed continuous variables with Wilcoxon rank-sum (or Kruskal-Wallis) tests or with ANOVAs when normally distributed. We performed chi-square (or Fisher’s exact) tests to analyze the between-group differences in categorical variables.

For the first aim, we used logistic regression models to assess the association between pre-transplant anti-LG3 level and DGF. We considered the possibility that the association between elevated anti-LG3 and DGF differed based on hypothermic machine perfusion by creating and testing an interaction term between these 2 variables. To identify confounders, we examined the association between each covariate listed above and both the exposure (high anti-LG3 antibodies) and the outcome (DGF). In the initial multivariable model, we included all the covariates with strong and moderate potential for confounding (see Supplementary Appendix SA1). Then, we simplified the multivariable model to avoid overfit by excluding one covariate at a time, starting with those having the highest *p*-values. Variables that were not associated with DGF in the initial multivariable model (*p*-value ≥0.05) and whose singular exclusion resulted in a <10% change in the point estimate for the main exposure (compared with the model including all covariates) were withdrawn from the final multivariable model. To ensure more thorough adjustment for confounders, we also fit a logistic regression model to identify the factors associated with the use of hypothermic perfusion. We then forced in the final model for DGF 2 variables that were independently associated with the use of hypothermic perfusion, while the 2 others were already included.

For the second aim, we fit a Fine and Gray proportional subdistribution hazards multivariate regression model for competing-risks to assess whether elevated pre-transplant anti-LG3 (dichotomized at the last quartile) was associated with graft failure, accounting for death as a competing risk. Patients who were lost to follow-up before the end of the study period were censored without an event at the date they were lost to follow-up. We assessed effect modification by including an interaction between anti-LG3 and DGF in the model. A backward selection approach was used to select covariables included in the final multivariable model. We performed a sensitivity analysis excluding 3 patients who had primary graft non-function. There were no missing values for the primary exposures or outcomes. Missing values for covariates were handled either by imputation (the mean for continuous variables) or by creating a “no or unknown” category for categorical variables.

## Results

### Pre-Transplant anti-LG3 are Associated With Delayed Graft Function When the Donor Kidney is Transported in Cold Preservation Solution, but not When the Kidney is Placed on Hypothermic Perfusion Machine

**FIGURE 1:**
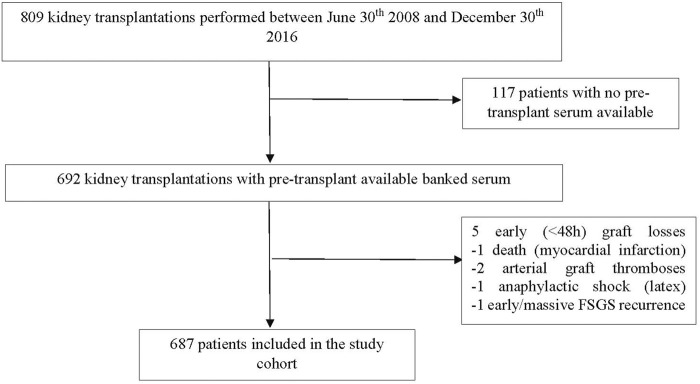
Patient flow chart.

Amongst the 809 patients who underwent kidney transplantation at the 2 centers during the study period, 687 were included in the analysis for the association between pre-transplant anti-LG3, DGF and graft survival (Figure 1). The reason for exclusion was the absence of available pre-transplant serum to test, which occurred at the inception of our biobank in 1 center for logistical reasons. Table 1 shows recipient, donor and procedure-related characteristics of patients who had pre-transplant sera available, stratified by anti-LG3 level. DGF occurred in 265 (39%) patients. The number of missing values for covariates are described at the bottom of Table 1. Patients who had high pre-transplant anti-LG3 antibodies were more likely to be of African descent, have diabetic nephropathy as a cause of CKD and have a positive pre-transplant CMV serology. Patients with high pre-transplant anti-LG3 had a slightly longer time on dialysis prior to transplant than those without pre-transplant anti-LG3. There was no association between elevated anti-LG3 antibodies and autoimmune disease as a cause of CKD, nor did we observe an association between anti-LG3 and pre-transplant anti-HLA antibodies or factors associated with their development such as transfusions, previous transplantations or pregnancies.

**TABLE 1:** Recipient and donor characteristics in kidney transplant recipients with pre-transplant sera available stratified by anti-LG3 level (*n* = 687 unless specified otherwise).

Characteristics	High anti-LG3 (n = 172)	Low anti-LG3 (n = 515)	*p*-value
Recipient			
Mean age at transplantation in years (SD)	52 (14)	51 (13)	0.67
Male sex, n (%)	112 (65)	315 (61)	0.35
African American race, n (%)	25 (15)	29 (6)	0.01
Mean body mass index kg/m^2^ (SD)	26 (5)	26 (4)	0.29
Cause of chronic kidney disease, n (%)			
Glomerular diseases	55 (32)	187 (36)	0.30
Diabetes	36 (21)	69 (13)	0.02
Hypertension/vascular	17 (10)	54 (10)	0.82
Polycystic kidney diseases	25 (15)	94 (18)	0.27
Autoimmune diseases	9 (5)	24 (5)	0.76
Other or unknown	30 (17)	87 (17)	0.71
Median time on dialysis pre-transplant in months, (IQR)	29 (10–55)	26 (1–49)	0.07
Positive CMV serology, n (%)	107 (62)	235 (46)	0.01
Pretransplant diabetes, n (%)	47 (27)	111 (22)	0.12
Coronary artery disease at transplantation, n (%)	34 (20)	85 (17)	0.33
Active smoking at transplantation, n (%)	29 (17)	69 (13)	0.26
Statin use at transplantation, n (%)	94 (55)	276 (54)	0.81
ACE inhibitor/angiotensin-2 blocker use at transplantation, n (%)	82 (48)	216 (42)	0.19
Median pre-transplant panel reactive antibodies (IQR)	0 (0–0)	0 (0–0)	0.51
Median peak historical panel reactive antibodies (IQR)	0 (0–4)	0 (0–5)	0.99
First transplantation, n (%)	157 (91)	456 (89)	0.32
HLA mismatches, n (%)			
0–2	25 (15)	114 (22)	0.03
3–4	93 (54)	255 (50)	0.30
5–6	54 (31)	146 (28)	0.45
Previous transfusions, n (%)	75 (44)	212 (41)	0.57
Previous pregnancies, n (%)	43 (25)	147 (29)	0.37
Induction^a^			
Thymoglobulin, n (%)	47 (27)	115 (22)	0.18
Intravenous immunoglobulin, n (%)	14 (8)	28 (5)	0.20
Plasma exchange, n (%)	2 (1)	1 (0.4)	0.73
Maintenance immunosuppression, n (%)			
Tacrolimus and mycophenolate mofetil	166 (97)	496 (96)	0.90
Cyclosporine and mycophenolate mofetil	4 (2)	11 (2)	0.88
Donor			
Living donor, n (%)	38 (22)	123 (24)	0.63
Deceased donor			
Neurological determination of death, n (%)	112 (65)	336 (65)	0.98
Donor after cardiocirculatory arrest, n (%)	22 (13)	56 (11)	0.49
Mean age in years, (SD)	49 (15)	49 (15)	0.85
Male sex, n (%)	92 (53)	271 (53)	0.84
Mean height in meters (SD)	1.68 (0.10)	1.68 (0.10)	0.82
Positive CMV serology, n (%)	72 (42)	190 (37)	0.25
Hypertension, n (%)	42 (24)	112 (22)	0.47
Diabetes, n (%)	13 (8)	39 (8)	1.00
Tobacco history, n (%)	88 (51)	273 (53)	0.70
Donor vascular disease, n (%)	20 (12)	41 (8)	0.14
Mean terminal serum creatinine in µmol/L (SD)	71 (37)	70 (45)	0.73
Procedure			
Median total ischemic time in hours, (IQR)	9 (5–13)	9 (5–15)	0.40
Use of hypothermic perfusion, n (%)	57 (33)	162 (31)	0.68
Center 1, n (%)	88 (51)	267 (52)	0.88
Early post-transplant course			
Delayed graft function, n (%)	72 (42)	193 (37)	0.31

^a^ One patient in the group low anti-LG3 received induction with alemtuzumab.

Missing data: *Recipient:* pre-transplant transfusion (*n* = 1), pre-transplant pregnancies (*n* = 2), smoking history (*n* = 6), *Donor:* terminal creatinine (*n* = 27), CMV serology (*n* = 3), height and weight (*n* = 2), hypertension (*n* = 27), diabetes (*n* = 33), peripheral vascular disease (*n* = 41), smoking history (*n* = 21); *Procedure:* total ischemic time (*n* = 12), use of hypothermic pump (*n* = 97).

SD, standard deviation; IQR, interquartile range.

While the use of hypothermic perfusion during organ transportation was left to the discretion of transplant surgeons recovering or transplanting the kidney, we identified some associations between clinical characteristics of the donors/recipients and use of hypothermic pump (Supplementary Table S1). In a multivariable analysis, its use differed by center, was higher in recent years and in donors with diabetes and in donors after cardiocirculatory death (Supplementary Table S2). The use of hypothermic perfusion machine modified the association between high pre-transplant anti-LG3 levels and DGF. In multivariable analyses (Table 2), pre-transplant anti-LG3 values in the upper distribution quartile were independently associated with DGF (odds ratio (OR): 1.75, 95% confidence interval 1.02–3.00, *p* = 0.04) when the donor kidney was transported in static cold storage (*n* = 468). When the kidney was placed on hypothermic machine perfusion (*n* = 219), there was no association between anti-LG3 and DGF (OR: 0.78, 95% CI 0.44–1.37, *p* = 0.38). Recipient diabetes, longer time on dialysis prior to transplantation, use of thymoglobulin, older donor age, neurologically deceased or deceased after cardiocirculatory arrest versus living donor, donor serum creatinine >120 µmol/L were all associated with a higher risk of DGF. Use of renin-angiotensin system blockers at the time of transplantation and higher donor height were associated with a lower risk of DGF, while there was a center effect in the incidence of DGF. Univariable analyses are presented in Supplementary Table S3 and the initial multivariable model (before simplification) is presented in Supplementary Table S4.

**TABLE 2:** Associations between recipient, donor and procedure characteristics and DGF in final multivariable analyses (*n* = 687).

Recipient/Donor/Procedure characteristics	Univariable odds ratio (95% CI^a^)	*p*-value	Multivariable odds ratio (95% CI^a^)	*p*-value
Pre-transplant elevated anti-LG3 antibodies^b^				
In cold static storage	1.58 (1.03, 2.39)	0.04	1.75 (1.02, 3.00)	0.04
Placed on a hypothermic perfusion machine	0.65 (0.33, 1.29)	0.22	0.78 (0.44, 1.37)	0.38
Recipient age at transplant (per 10 years higher)	1.17 (1.04, 1.31)	<0.01	0.98 (0.97, 1.00)	0.06
Recipient African American race (versus Caucasian)	1.80 (1.03, 3.14)	0.04	0.84 (0.39, 1.79)	0.65
Time on dialysis pre-transplant (per 1-month higher)	1.00 (1.00, 1.20)	<0.01	1.01 (1.01, 1.02)	<0.01
Recipient diabetes	2.34 (1.63, 3.36)	<0.01	2.14 (1.35, 3.39)	<0.01
Recipient positive CMV serology	1.28 (0.94, 1.74)	0.11	1.02 (0.68, 1.54)	0.92
Recipient ACE inhibitor/angiotensin-2 blocker use at transplantation	0.57 (0.42, 0.79)	<0.01	0.54 (0.37, 0.81)	<0.01
Previous transplantations	1.69 (1.04, 2.74)	0.03	1.61 (0.84, 3.10)	0.15
Thymoglobulin induction	2.34 (1.63, 3.35)	<0.01	2.24 (1.36, 3.69)	<0.01
Donor type (References neurologically deceased)				
Living donor	0.12 (0.06, 0.21)	<0.01	0.21 (0.11, 0.41)	<0.01
Donor after cardiac arrest	4.10 (2.37, 7.11)	<0.01	4.99 (2.62, 9.50)	<0.01
Donor age (per 10-year higher)	1.30 (1.17, 1.45)	<0.01	1.48 (1.28, 1.72)	<0.01
Donor height (per 10 cm higher)	0.82 (0.71, 0.95)	<0.01	0.75 (2.34, 15.63)	<0.01
Donor peripheral vascular disease	2.00 (1.18, 3.40)	0.01	0.96 (0.50, 1.87)	0.91
Donor diabetes	1.52 (0.86, 2.69)	0.15	0.94 (0.48, 1.85)	0.59
Donor terminal serum creatinine ≥120 umol/L	4.69 (2.05, 10.69)	<0.01	6.14 (2.37, 15.92)	<0.01
Center 1	0.31 (0.22, 0.42)	<0.01	0.38 (0.23, 0.63)	<0.01
Transplant date (per 1 year higher)	1.04 (0.97, 1.11)	0.26	1.03 (0.93, 1.13)	0.86

^a^ CI, confidence interval.

^b^ The *p*-value for the interaction between anti-LG3 and use of hypothermic pump is 0.02.

### High Pre-transplant anti-LG3 are Associated With a Higher Risk of Graft Failure in Patients Who Experienced DGF, but not in Those With Immediate Graft Function

**FIGURE 2:**
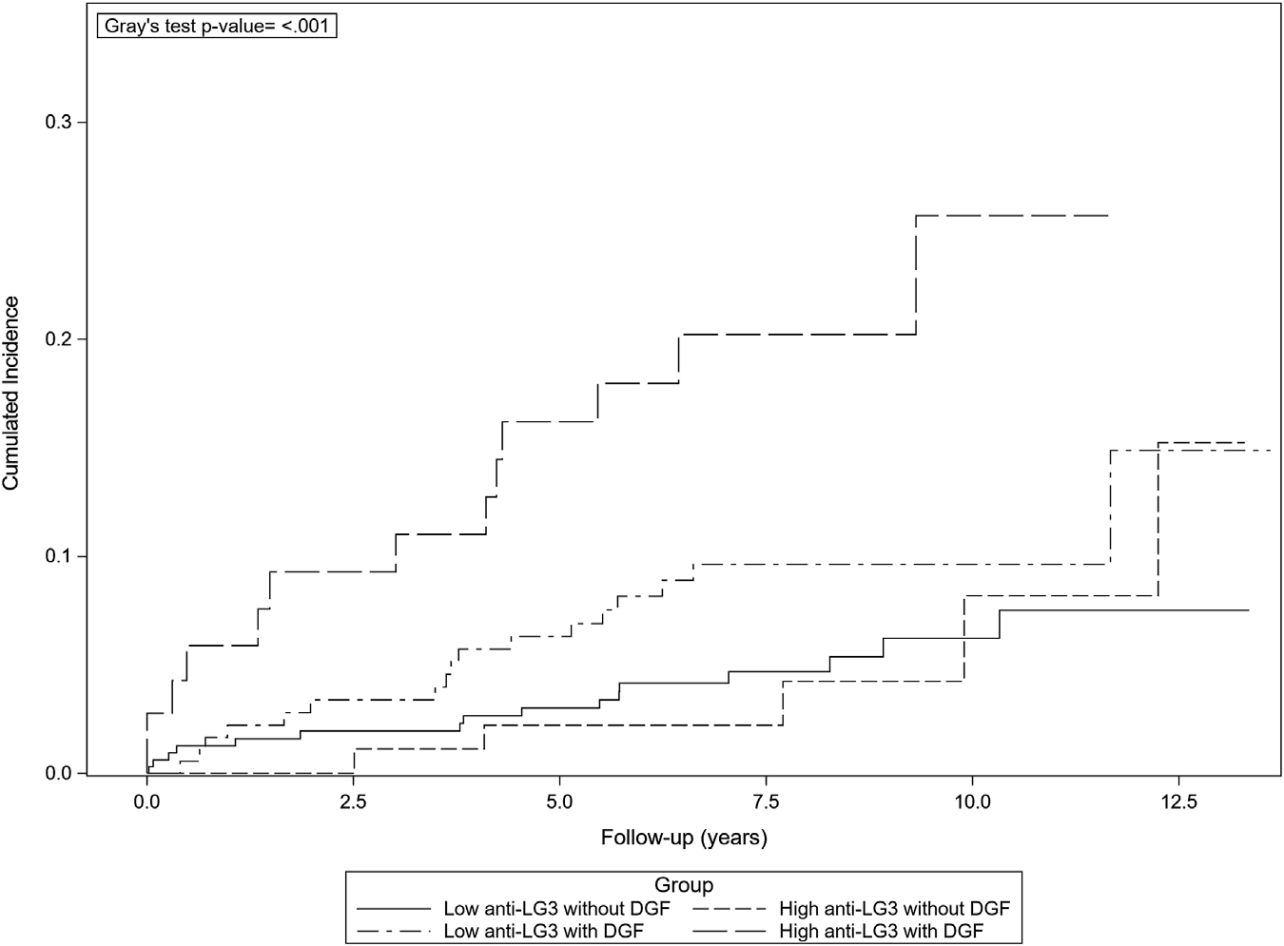
Cumulative incidence of death-censored graft loss by delayed graft function and anti-LG3 status. Patients with both high anti-LG3 and DGF show an increased risk of death-censored graft loss taking into account the competitive risk of death (*p* < 0.001).

We had previously shown that patients in patients with DGF, high pre-transplant anti-LG3 were associated with reduced eGFR 1 year post transplant, which was not the case in patients with immediate graft function (10). To verify whether this led to long-term poor outcomes, we examined the association between pre-transplant anti-LG3 and kidney graft survival in patients with and without DGF, accounting for the competing risk of death. Over a median follow-up of 6.9 years (interquartile range (IQR) 5.2–8.9 years), 93 patients died and 51 experienced graft loss (return to hemodialysis or retransplantation). The presence of DGF modified the association between anti-LG3 and graft survival. In patients with DGF, high anti-LG3 was associated with a higher risk of graft loss (subdistribution hazard ratio (SHR): 4.07, 95% CI: 1.80, 9.22), while this was not the case in patients with immediate graft function (SHR: 0.50, 95% CI 0.19, 1.29) (Table 3; Figure 2). Other factors that were associated with a higher risk of graft loss were younger recipient age, time on dialysis pre-transplant, number of HLA mismatches, donor age, donor female sex and higher donor serum creatinine, earlier vintage, transplant center, as well as active recipient smoking at the time of transplant and induction with intravenous immunoglubulins. Univariable analyses are presented in Supplementary Table S5. Removing the 3 patients with primary non-function did not modify the results.

**TABLE 3:** Associations between recipient, donor and procedure characteristics and graft survival in final multivariable analyses (n = 687).

Recipient/Donor/Procedure characteristics	Univariable SHR (95% CI^a^)	*p*-value	Multivariable SHR (95% CI^a^)	*p*-value
Pre-transplant elevated anti-LG3 antibodies^b^				
In patients with immediate graft function	0.97 (0.37, 2.58)	0.92	0.50 (0.19, 1.29)	0.15
In patients with delayed graft function	2.42 (1.19, 4.95)	0.01	4.07 (1.80, 9.22)	<0.01
Recipient age at transplant (per 1 year higher)	0.97 (0.95, 0.99)	0.01	0.96 (0.93, 0.98)	<0.01
Time on dialysis pre-transplant (per 1-month higher)	1.01 (1.01, 1.02)	<0.01	1.02 (1.01, 1.02)	<0.01
Active recipient smoking at transplantation	2.68 (1.50, 4.80)	<0.01	3.66 (2.05, 6.56)	<0.01
Recipient prior history of coronary artery disease	0.40 (0.15, 1.11)	0.08	0.26 (0.08, 0.83)	0.02
Center 1	0.64 (0.37, 1.12)	0.12	0.54 (0.29, 0.99)	0.05
Transplant date (per 1 year higher)	0.91 (0.81, 1.02)	0.09	0.87 (0.76, 0.99)	0.04
Induction with intravenous immunoglobulins	1.87 (0.79, 4.44)	0.15	4.07 (1.44, 11.50)	<0.01
Donor age (per 10-year higher)	1.22 (0.97, 1.54)	0.09	1.56 (1.19, 2.04)	<0.01
Female donor sex	1.64 (0.94, 2.85)	0.08	2.04 (1.17, 3.53)	0.01
Donor serum creatinine (per 10 umol/L higher)	1.03 (1.00, 1.06)	0.10	1.07 (1.04, 1.10)	<0.01
HLA mismatches (per 1 mismatch higher)	1.19 (0.97, 1.46)	0.10	1.35 (1.04, 1.76)	0.02

^a^ SHR: subdivision hazard ratio, CI: confidence interval.

^b^ The *p*-value for the interaction between anti-LG3 and DGF is 0.001.

## Discussion

The characterization of novel biomarkers and/or potential therapeutic targets that could prevent the occurrence of DGF and its adverse impact on long-term allograft outcomes could represent significant advances in the field of kidney transplantation. Here, we found that pre-transplant anti-LG3 antibodies were associated with an increased risk of DGF, but only when the organ was placed in static cold storage during transportation. When the organ was placed on hypothermic perfusion machine, this association was no longer seen. We also examined whether DGF, as a proxy for severe IRI, was a permissive factor for the adverse impact of anti-LG3 on graft survival to occur. We hence extended findings from our previous work by showing that in patients with DGF, but not in those with immediate graft function, pre-transplant anti-LG3 levels are associated with a higher risk of graft loss.

Taken together, our findings are consistent with our initial hypothesis, namely that IRI favors the exposure of cryptic autoantigens, such as LG3, from the graft microvasculature setting in motion autoantibody-dependent microvascular injury and rarefaction. We showed previously that renal IRI in mice leads to increased circulating levels of apoptotic exosome-like vesicles bearing LG3 (23). When the allograft is transplanted into a recipient with elevated anti-LG3 antibodies, these antibodies may bind to their antigenic targets if the vasculature is already stressed allowing for LG3 release or exposure. This is turn would favor antibody-antigen interactions and complement activation, therefore promoting microvascular damage and fibrosis leading to decreased graft survival. Indeed, injection of anti-LG3 antibodies in a murine model of renal IRI led to microvascular rarefaction, complement deposition and enhanced renal fibrosis while this was not the case when the mice had not undergone renal IRI (10). Other autoantibodies have also been reported to promote tissue inflammation and injury in the presence of IRI. Angiotensin II type 1 receptor (AT1R) antibodies alter the vascular reactivity of renal arteries, but only in the presence of ischemia or allogeneic transplantation (11). Infusion of autoantibodies against myosin lead to syngeneic heterotopic heart transplant failure in mice when administered at the time of IRI, but not when administered later on (24). Transfer of autoantibodies to K-alpha-1-tubulin and Collagen V leads to inflammation and tissue injury in a model of syngeneic orthotopic lung transplantation while sham treated mice who received these autoantibodies developed no lesion (12). Human studies cannot reproduce the level of details obtained in experimental models. Nevertheless, one can hypothesize that in the presence of hypothermic machine perfusion, a technique that reduces endothelial injury and improves microcirculatory flow in animal models of kidney transplantation (19), the exposure of cryptic autoantigens such as LG3 and potential for anti-LG3 interaction and graft injury may be diminished. Hence, the results from the present study support a role for IRI as a permissive factor for anti-LG3 to adversely affect the kidney transplant.

DGF is associated with reduced long-term kidney graft survival in most studies (1). Nevertheless, the outcome of patients with DGF is not uniformly unfavorable and can be modulated by factors such as donor type and donor age (2,3). While biomarkers such as kidney injury molecule-1 and neutrophil gelatinase-associated associated lipocalin are associated with DGF, they show no association with subsequent graft function in patients with DGF (25). These biomarkers, which evaluate tubular epithelial cell injury or inflammation, may not be reflective of microvascular injury, which plays a predominant role in predicting loss of renal function on the long-term (6,7). We recently observed in caspase-3 deficient mice that show enhanced early epithelial injury after IRI but preservation of PTC integrity, that microvascular injury, but not early tubular damage, predicts renal fibrosis and progressive loss of renal function (6). We also showed, in renal transplant patients with DGF, that loss of PTC density in the first year after transplantation is associated with eGFR 1 and 3 years post-transplantation (4). These observations are in line with multiple studies establishing a link between microvascular damage, renal fibrosis and progressive renal dysfunction (4,5,7,10,15,26–28). The identification of factors regulating microvascular injury at the time of transplantation should lead to a better capacity to predict and prevent long-term renal dysfunction.

In the present study, other factors associated with DGF and graft survival were those observed in many previous studies (20,21,29). Although RAS blockers are discontinued at the time of transplantation in our centers, we had hypothesized that the biological effect of long-acting RAS blockers could have increased the risk of DGF (30). In contrast to our *a priori* expectations, use of RAS blockers were associated with a decreased risk of DGF. Although prior studies have shown a null (31) or protective impact of perioperative RAS system blockers on short-term graft function (32) and long-term allograft survival (33,34), many clinicians may fear hyperkalemia or hemodynamic deterioration in kidney function in the early post-transplant period and discontinue these medications. Intervention studies examining the benefit of these medications at the time of transplant are needed to clarify this issue. Induction with intravenous immunoglobulins was associated with a higher risk of graft failure, which is probably due to the fact that this induction was reserved to patients perceived at the highest immunological risk by their transplant physicians.

In addition to increasing length and cost of hospital stay for kidney transplantation (35), DGF is associated with reduced long-term kidney graft survival in most studies (1). Hence, strategies that decrease its occurrence should be of clinical benefit. The use of hypothermic perfusion has been shown to lower the incidence of DGF in multiple studies (36). Nevertheless, its use varies by center in our jurisdiction (37), which may be due to logistic reasons as well as to the absence of clearly demonstrated benefits to kidney graft survival (36). Measuring pre-transplant anti-LG3 may be a strategy to identify recipients who would derive the greatest benefit from hypothermic machine perfusion of the donor kidney. Other strategies such as plasma exchange could also be tested in patients with high anti-LG3 antibodies.

Our study has certain limitations. First, it was performed in 2 University-affiliated centers, with a majority of Caucasian patients at low immunological risk, which may affect its generalizability. Also, given the observational and retrospective nature of the study, and the absence of early post-transplant biopsies, we can only speculate as to the mechanisms by which anti-LG3 adversely affect allograft outcomes and report associations which may or may not be causal. Moderate elevations in anti-LG3, for instance when anti-LG3 was stratified in tertiles, did not lead to a significant increase in the risk of DGF, but remained associated with kidney graft survival (data not shown). Hence, the levels at which anti-LG3 can be considered deleterious may vary by outcome. Last, when we were unable to retrieve information on the use of hypothermic pump from the chart, we classified this in the ‘no or unknown’ category. Although may have resulted in misclassification, this is likely to have been non-differential, resulting in bias towards observing no difference by machine perfusion status.

In conclusion, the present study supports the notion that IRI represents a permissive factor for anti-LG3 to exert a negative effect on the allograft. High anti-LG3 levels are associated with a higher risk of DGF in kidneys exposed to cold storage but not in kidneys place on hypothermic pump perfusion. Favoring the use of hypothermic perfusion for organs to be transplanted to candidates with high anti-LG3 levels may offer a therapeutic strategy to prevent DGF and extend renal allograft survival.

## Data Availability

The datasets presented in this article are not readily available because the author is willing to share the raw data only if this is allowed by the institutional Ethics Committee of Centre Hospitalier de l’Université de Montréal. Requests to access the datasets should be directed to heloise.cardinal.chum@ssss.gouv.qc.ca.

## Abbreviations

DCD, Donation after cardio-circulatory death; DGF, Delayed graft function; DSA, Donor-specific antibodies; eGFR, Estimated glomerular filtration rate; HLA, Human leukocyte antigen; HR, Hazard ratio; IRI, Ischemia-reperfusion injury; IQR, Interquartile range; OR, Odds ratio; PTC, Peritubular capillary.
