# Supplementary material for: Hypothermic Perfusion Modifies the Association Between Anti-LG3 Antibodies and Delayed Graft Function in Kidney Recipients
Source: Transpl Int. 2023 Feb 20;36:10749. doi: 10.3389/ti.2023.10749 (PMC9986256; doi:10.3389/ti.2023.10749)
Supplement: Supplementary file 1 [file Table5.pdf]

**Supplementary Table 5.** Associations between recipient, donor and procedure characteristics and graft survival in univariable analyses (n=687).		
Recipient/ Donor/ Procedure characteristics	Subdivision Hazard Ratio (95 % CI)	p-value
Pre-transplant elevated anti-LG3 antibodies		
In patients with immediate graft function	0.97 (0.37, 2.58)	0.96
In patients with delayed graft function	2.42 (1.19, 4.95)	0.02
Recipient age at transplant (per 1 year higher)	0.97 (0.95, 0.99)	0.01
Recipient female sex (versus male)	1.12 (0.64, 1.95)	0.69
African American race	2.45 (1.19, 5.04)	0.02
Transplant date (per 1 year higher)	0.91 (0.81, 1.02)	0.09
Recipient body mass index (per 1 unit higher)	0.68 (0.30, 1.50)	0.34
Cause of CKD		
Glomerular diseases (ref all other causes)	0.67 (0.37, 1.23)	0.20
Diabetes (ref all other causes)	0.74 (0.32, 1.73)	0.49
Time on dialysis pre-transplant (per 1 month higher)	1.01 (1.01, 1.02)	<0.01
Recipient positive CMV serology	1.72 (0.98, 3.03)	0.06
Recipient diabetes	0.53 (0.24, 1.17)	0.12
Recipient coronary artery disease at transplantation	0.40 (0.15, 1.11)	0.08
Active smoking at transplantation (reference never smoked)	2.68 (1.50, 4.80)	<0.01
Pre-transplant PRA	1.01 (1.00, 1.03)	0.17
Peak historical PRA	1.01 (1.00, 1.02)	0.02
Previous transplantations	1.91 (0.92, 3.96)	0.08
Number of HLA mismatches (per 1 mismatch higher)	1.19 (0.97, 1.46)	0.10
Previous transfusions	1.64 (0.95, 2.84)	0.08
Previous pregnancies	1.27 (0.71, 2.27)	0.42
Thymoglobulin induction	2.12 (1.21, 3.72)	0.01
Donor type (reference neurologically deceased)		
Living donor	0.56 (0.27, 1.19)	0.13
Donor after cardiac arrest	0.52 (0.16, 1.67)	0.27
Donor age (per 10-year higher)	1.22 (0.97, 1.54)	0.09
Female donor sex (versus male)	1.64 (0.94, 2.85)	0.08

Donor height (per 10 cm higher)	0.81 (0.67, 0.99)	0.04
Donor positive CMV serology	1.63 (0.94, 2.83)	0.08
Donor hypertension	1.70 (0.94, 3.07)	0.08
Donor diabetes	0.55 (0.13, 2.27)	0.41
Donor positive smoking history (versus negative or unknown)	1.00 (0.58, 1.74)	0.99
Donor peripheral vascular disease	0.93 (0.34, 2.57)	0.89
Donor terminal serum creatinine (per 10 umol/L higher)	1.03 (1.00, 1.06)	0.10
Total ischemic time (per 1 hour higher)	1.03 (0.99, 1.08)	0.18
Use of hypothermic perfusion machine	0.68 (0.36, 1.32)	0.25
Center 1	0.64 (0.37, 1.12)	0.12
