# Supplementary material for: Hypothermic Perfusion Modifies the Association Between Anti-LG3 Antibodies and Delayed Graft Function in Kidney Recipients
Source: Transpl Int. 2023 Feb 20;36:10749. doi: 10.3389/ti.2023.10749 (PMC9986256; doi:10.3389/ti.2023.10749)
Supplement: Supplementary file 2 [file Table4.pdf]

Supplementary Table 4. Associations between recipient, donor and procedure characteristics and DGF in initial multivariable analyses (n=687).

Recipient/ Donor/ Procedure characteristics	Univariable Odds ratio* (95 % CI)	p-value	Initial Multivariable Odds ratio* (95 % CI)	p-value
Pre-transplant elevated anti-LG3 antibodies§				
In cold static storage	1.58 (1.03, 2.39)	0.04	1.75 (1.00, 3.05)	0.05
Placed on a hypothermic perfusion machine	0.65 (0.33, 1.29)	0.22	0.83 (0.47, 1.49)	0.54
Recipient age at transplant (per 10 year higher)	1.17 (1.04, 1.31)	<0.01	0.82 (0.68, 0.98)	0.03
Recipient African American race (versus Caucasian)	1.80 (1.03, 3.14)	0.04	0.90 (0.41, 1.95)	0.78
Recipient body mass index ≥30 kg/m^2	1.52 (1.04, 2.22)	0.03	1.29 (0.78, 2.13)	0.32
Time on dialysis pre-transplant (per 1-month higher)	1.00 (1.00, 1.20)	<0.01	1.01 (1.01, 1.02)	<0.01
Recipient diabetes	2.34 (1.63, 3.36)	<0.01	1.89 (1.15, 3.12)	0.01
Recipient positive CMV serology	1.28 (0.94, 1.74)	0.11	1.04 (0.68, 1.59)	0.86
Recipient coronary artery disease at transplantation	2.04 (1.37, 3.03)	<0.01	1.19 (0.70, 2.05)	0.52
Recipient ACE inhibitor/angiotensin-2 blocker use at transplantation	0.57 (0.42, 0.79)	<0.01	0.50 (0.33, 0.76)	<0.01
Pre-transplant PRA > 50%	2.42 (0.68, 8.66)	0.17	1.00 (0.98, 1.02)	0.89
Peak historical PRA > 50%	1.98 (0.98, 4.00)	0.06	1.00 (0.99, 1.02)	0.43
Previous transplantations	1.69 (1.04, 2.74)	0.03	1.85 (0.90, 3.77)	0.09
Previous transfusions	1.85 (1.35, 2.52)	<0.01	0.69 (0.44, 1.09)	0.11
Thymoglobulin induction	2.34 (1.63, 3.35)	<0.01	2.09 (1.24, 3.52)	<0.01
Donor type (reference neurologically deceased)				
Living donor	0.12 (0.06, 0.21)	<0.01	0.20 (0.09, 0.45)	<0.01
Donor after cardiac arrest	4.10 (2.37, 7.11)	<0.01	5.67 (2.87, 11.20)	<0.01
Donor age (per 10-year higher)	1.30 (1.17, 1.45)	<0.01	1.57 (1.34, 1.84)	<0.01
Donor height (per 10 cm higher)	0.82 (0.71, 0.95)	<0.01	0.77 (0.64, 0.92)	<0.01
Donor hypertension	1.97 (1.37, 2.82)	<0.01	0.81 (0.49, 1.33)	0.41
Donor positive smoking history	1.72 (1.26, 2.35)	<0.01	0.82 (0.54, 1.26)	0.36
Donor peripheral vascular disease	2.00 (1.18, 3.40)	0.01	1.08 (0.54, 2.17)	0.82
Donor terminal serum creatinine ≥120 umol/L	4.69 (2.05, 10.69)	<0.01	6.48 (2.49, 16.89)	<0.01
Total ischemic time (per 1-hour higher)	1.08 (1.05, 1.11)	<0.01	1.02 (0.98, 1.06)	0.43
Center 1	0.31 (0.22, 0.42)	<0.01	0.33 (0.20, 0.56)	<0.01

§ The p-value for the interaction between anti-LG3 and use of hypothermic pump is 0.02
