# Supplementary material for: Hypothermic Perfusion Modifies the Association Between Anti-LG3 Antibodies and Delayed Graft Function in Kidney Recipients
Source: Transpl Int. 2023 Feb 20;36:10749. doi: 10.3389/ti.2023.10749 (PMC9986256; doi:10.3389/ti.2023.10749)
Supplement: Supplementary file 3 [file Table1.pdf]

Supplementary Table 1. Recipient and donor characteristics in kidney transplant recipients with pre-transplant sera available stratified by use of hypothermic machine perfusion

Characteristics	Hypothermic perfusion (n=219)	Cold static storage (n=468)	p-value
<u>Recipient</u>			
High pre-transplant anti-LG3	57 (26)	115 (25)	0.68
Mean age at transplantation in years (SD)	53 (13)	51 (14)	0.06
Male sex, n (%)	146 (67)	281 (60)	0.09
African American race, n (%)	20 (9)	34 (7)	0.39
Mean body mass index kg/m^2 (SD)	26 (4)	26 (5)	0.61
Cause of chronic kidney disease, n (%)			
Glomerular diseases	79 (36)	163 (35)	0.75
Diabetes	31 (14)	74 (16)	0.57
Hypertension/vascular	19 (9)	52 (11)	0.33
Polycystic kidney diseases	40 (18)	79 (17)	0.66
Autoimmune diseases	7 (3)	26 (6)	0.18
Median time on dialysis pre-transplant in months, (IQR)	38 (15-59)	21 (0-46)	<0.01
Positive CMV serology, n (%)	124 (57)	218 (47)	0.01
Pretransplant diabetes, n (%)	46 (21)	112 (24)	0.40
Coronary artery disease at transplantation, n (%)	38 (17)	81 (17)	0.99
Active smoking at transplantation, n (%)	28 (13)	70 (15)	0.45
Statin use at transplantation, n (%)	114 (52)	256 (55)	0.52
ACE inhibitor/ angiotensin-2 blocker at transplant, n (%)	92 (42)	206 (44)	0.62
Median pre-transplant panel reactive antibodies (IQR)	0 (0-0)	0 (0-0)	0.58
Median peak historical panel reactive antibodies (IQR)	0 (0-4)	0 (0-5)	0.52
First transplantation, n (%)	196 (90)	417 (89)	0.88
HLA mismatches, n (%)			
0-2	33 (15)	106 (23)	0.02
3-4	104 (48)	244 (52)	0.26
5-6	82 (37)	118 (25)	0.01
Previous transfusions, n (%)	89 (41)	198 (42)	0.68
Previous pregnancies, n (%)	52 (24)	138 (29)	0.12
Induction with thymoglobulin, n (%)	61 (28)	101 (22)	0.07

Donor			
Deceased donor			
Neurological determination of death, n (%)	140 (64)	308 (66)	0.63
Donor after cardiocirculatory arrest, n (%)	32 (15)	46 (10)	0.07
Expanded criteria donor, n (%)	94 (43)	177 (38)	0.20
Mean age in years, (SD)	50 (14)	49 (16)	0.85
Male sex, n (%)	112 (51)	251 (53)	0.54
Mean height in meters (SD)	1.68 (0.1)	1.69 (0.1)	0.42
Positive CMV serology, n (%)	89 (59)	173 (63)	0.36
Hypertension, n (%)	48 (22)	106 (23)	0.83
Diabetes, n (%)	22 (10)	30 (6)	0.09
Tobacco history, n (%)	108 (48)	253 (54)	0.25
Donor vascular disease, n (%)	13 (6)	48 (10)	0.06
Mean terminal serum creatinine in µmol/L (SD)	66 (25)	72 (48)	0.11
Procedure			
Median total ischemic time in hours, (IQR)	9 (5-14)	9 (5-15)	0.38
Center 1, n (%)	190 (87)	165 (35)	<0.01
Median transplant date (IQR)	17/2/2015 (26/11/2013-30/12/2015)	6/10/2012 (6/11/2010-12/7/2014)	<0.01
