# Supplementary material for: Hypothermic Perfusion Modifies the Association Between Anti-LG3 Antibodies and Delayed Graft Function in Kidney Recipients
Source: Transpl Int. 2023 Feb 20;36:10749. doi: 10.3389/ti.2023.10749 (PMC9986256; doi:10.3389/ti.2023.10749)
Supplement: Supplementary file 4 [file Table2.pdf]

Supplementary Table 2. Donor, recipient and procedure characteristics associated with use of hypothermic perfusion machine during organ transportation, n=687

Recipient/ Donor/ Procedure characteristics	Multivariable Odds Ratio (95 % CI*)	p-value
Recipient age at transplant (per 1 year higher)	1.01 (0.99, 1.04)	0.21
Female recipient sex	0.62 (0.26, 1.52)	0.30
Time on dialysis pre-transplant (per 1-month higher)	1.01 (1.00, 1.01)	0.08
Recipient positive CMV serology	0.98 (0.58, 1.65)	0.93
Prior pregnancies	1.13 (0.42, 3.00)	0.81
HLA mismatch (ref 3-4)	1.00	
0-2	0.74 (0.35, 1.57)	0.43
5-6	1.05 (0.59, 1.85)	0.88
Transplant year (per 1-year higher)	2.10 (1.77, 2.47)	<0.01
Thymoglobulin induction	1.16 (0.62, 2.15)	0.65
Donor after cardiocirculatory death	2.98 (1.25, 7.08)	0.01
Donor diabetes	5.52 (2.13, 14.32)	<0.01
Donor peripheral vascular disease	0.67 (0.26, 1.72)	0.82
Donor terminal serum creatinine (per 10 umol/L higher)	1.00 (1.00, 1.00)	0.99
Center 1	46.72 (23.04, 94.73)	<0.01

*CI: confidence interval
