# Supplementary material for: Hypothermic Perfusion Modifies the Association Between Anti-LG3 Antibodies and Delayed Graft Function in Kidney Recipients
Source: Transpl Int. 2023 Feb 20;36:10749. doi: 10.3389/ti.2023.10749 (PMC9986256; doi:10.3389/ti.2023.10749)
Supplement: Supplementary file 5 [file Table3.pdf]

Supplementary Table 3. Associations between recipient, donor and procedure characteristics and DGF in univariable analyses (n=687).

Recipient/ Donor/ Procedure characteristics	Odds ratio (95 % CI)	p-value
Pre-transplant elevated anti-LG3 antibodies§		
In cold static storage	1.58 (1.03, 2.39)	0.04
Placed on a hypothermic perfusion machine	0.65 (0.33, 1.29)	0.22
Recipient age at transplant (per 10 year higher)	1.17 (1.04, 1.31)	<0.01
Recipient female sex (versus male)	0.99 (0.72, 1.36)	0.96
Recipient African American race (versus Caucasian)	1.80 (1.03, 3.14)	0.04
Recipient body mass index (per 1 unit higher)	1.52 (1.04, 2.22)	0.03
Cause of CKD		
Other or unknown (reference)		
Glomerular diseases	0.90 (0.60, 1.35)	0.60
Diabetes	1.95 (1.20, 3.18)	<0.01
Hypertension/vascular	1.63 (0.95, 2.81)	0.07
Polycystic kidney diseases	0.85 (0.51, 1.40)	0.52
Autoimmune diseases	0.86 (0.39, 1.89)	0.70
Time on dialysis pre-transplant (per 1 month higher)	1.00 (1.00, 1.20)	<0.01
Recipient positive CMV serology	1.28 (0.94, 1.74)	0.11
Recipient diabetes	2.34 (1.63, 3.36)	<0.01
Coronary artery disease at transplantation	2.04 (1.37, 3.03)	<0.01
Recipient smoking history (reference never smoked)		
Former smoker	1.04 (0.74, 1.44)	0.84
Active smoking at transplantation	1.19 (0.75, 1.89)	0.46
Recipient statin use at transplantation	0.91 (0.67, 1.24)	0.56
Recipient ACE inhibitor/angiotensin-2 blocker use at transplantation	0.57 (0.42, 0.79)	<0.01
Pre-transplant PRA > 50%	2.42 (0.68, 8.66)	0.17
Peak historical PRA > 50%	1.98 (0.98, 4.00)	0.06
Previous transplantations	1.69 (1.04, 2.74)	0.03
HLA mismatches (per 1 mismatch higher)	1.10 (0.99, 1.23)	0.08
Previous transfusions	1.85 (1.35, 2.52)	<0.01
Previous pregnancies	0.99 (0.70, 1.39)	0.96
Thymoglobulin induction	2.34 (1.63, 3.35)	<0.01
Donor type (reference neurologically deceased)		
Living donor	0.12 (0.06, 0.21)	<0.01
Donor after cardiac arrest	4.10 (2.37, 7.11)	<0.01
Donor age (per 10-year higher)	1.30 (1.17, 1.45)	<0.01

§ The p-value for the interaction between anti-LG3 and use of hypothermic pump is 0.03

Female donor sex (versus male)	1.00 (0.74, 1.36)	1.00
Donor height (per 10 cm higher)	0.82 (0.71, 0.95)	<0.01
Donor positive CMV serology	0.90 (0.66, 1.24)	0.51
Donor hypertension	1.97 (1.37, 2.82)	<0.01
Donor diabetes	1.52 (0.86, 2.69)	0.15
Donor vascular disease	2.00 (1.18, 3.40)	0.01
Donor positive smoking history (versus negative or unknown)	1.72 (1.26, 2.35)	<0.01
Donor terminal serum creatinine ≥120 umol/L	4.69 (2.05, 10.69)	<0.01
Total ischemic time (per 1 hour higher)	1.08 (1.05, 1.11)	<0.01
Use of hypothermic perfusion machine	0.62 (0.44, 0.87)	<0.01
Center 1	0.31 (0.22, 0.42)	<0.01
Transplant date (per 1 year higher)	1.04 (0.97, 1.11)	0.26
