# Supplementary material for: Hypothermic Perfusion Modifies the Association Between Anti-LG3 Antibodies and Delayed Graft Function in Kidney Recipients
Source: Transpl Int. 2023 Feb 20;36:10749. doi: 10.3389/ti.2023.10749 (PMC9986256; doi:10.3389/ti.2023.10749)
Supplement: Supplementary file 6 [file DataSheet1.pdf]

Rationale for inclusion of potential confounders in multivariable model for DGF

Aim 1: Association between pre-transplant anti-LG3 (exposure) and DGF (outcome)

Strong potential for confounding based on association with both the exposure and the outcome (p-value ≤0.15 for association with both exposure and outcome)

African American race

Diabetes as cause of CKD

Positive serology for CMV in the recipient

Wait time on dialysis prior to transplantation

Recipient diabetes

Donor history of vascular disease

Moderate potential for confounding based on association with either exposure <u>or</u> the outcome, but <u>not</u> with both (p-value ≤0.15 for association with either exposure or outcome)

Association with DGF (outcome) only

Recipient age

Recipient body mass index

Recipient history of coronary artery disease

Previous transplantations

Ace inhibitors/ARBs at transplantation

Peak PRA >50%

Prior transfusions

Use of thymoglobulin as induction

Donor height

Donor type (living, neurologically deceased, after cardiocirculatory arrest)

Donor smoking history

Donor age

Donor terminal creatinine

Total ischemic time

Center

Use of hypothermic perfusion pump

Association with high anti-LG3 (exposure) only

None
